# Food safety dynamics in urban wet markets: knowledge, attitudes, and practices of poultry vendors in Nairobi, Kenya

**DOI:** 10.1371/journal.pone.0356776

**Published:** 2026-08-24

**Authors:** Lolyne Gatwiri, Catherine Kunyanga, Lucy Njue, Maya Nadimpalli, Alex Ndiritu, Angela Mwangi

**Affiliations:** 1 Department of Food Science, Nutrition and Technology, University of Nairobi, Nairobi, Kenya; 2 Gangarosa Department of Environmental Health, Emory University, Atlanta, Georgia, United States of America; 3 Department of Public Health, University of Kabianga, Kericho, Kenya; 4 Department of Biological and Life Sciences, The Technical University of Kenya, Nairobi, Kenya; University of Georgia, UNITED STATES OF AMERICA

## Abstract

Wet markets are crucial settings along poultry food supply chains, where lapses in food safety may facilitate the entry and spread of antimicrobial-resistant (AMR) microorganisms, such as *Escherichia coli* and *Salmonella enterica,* from poultry to humans. This cross-sectional study assessed food safety knowledge, attitudes, and practices among 141 poultry vendors in Burma Market, the largest poultry wet market in Nairobi. Food safety knowledge was low among 70.9% of vendors; 59.6% displayed negative attitudes, and 51.1% demonstrated poor practices. Younger vendors (18–24 years) had lower odds of good food safety knowledge (OR = 0.270, 95% CI = 0.029–2.197, p = 0.044) and practices (OR= 0.193, 95% CI = 0.136–2.327, p = 0.043) compared to those aged 45 + years. Female vendors had lower odds of good food safety practices than men (OR = 0.411, 95% CI = 0.182–0.926, p = 0.032). Household income was associated with food safety outcomes, where middle-income vendors (USD 154–231) demonstrated higher knowledge but poorer attitudes (OR= 0.335, 95% CI = 0.125–0.898, p = 0.030) compared to higher earners USD 232). In contrast, low-income vendors (USD 154.67) had lower odds of good practices (OR = 0.254, p = 0.046). Positive attitudes showed higher odds of good food safety practices (OR=2.196, 95% CI = 0.998–4.834, p = 0.049). The findings in this study suggest that food safety practices among poultry vendors in urban wet markets are influenced not only by knowledge but also by vendor attitudes and socioeconomic factors. By understanding the drivers of food safety behavior in an urban Kenyan wet market, this study provides new evidence that structural and behavioral factors may play a greater role in shaping hygiene practices than knowledge alone. Interventions aimed at improving food safety in these settings should therefore move beyond training-focused approaches and also strengthen risk perception while improving access to affordable hygiene infrastructure.

## Introduction

Food safety is a critical global public health concern, with nearly one in ten people worldwide falling ill each year after consuming contaminated food, resulting in approximately 420,000 deaths [1]. In low- and middle-income countries (LMICs), these risks are amplified in wet markets, where live poultry are commonly sold and slaughtered on site and where there is weak regulatory oversight on hygiene and practices [2]. Global population growth is projected to reach approximately 9.7 billion people by 2050, increasing the demand for affordable sources of animal protein [3]. Poultry has become one of the most important protein sources worldwide, accounting for nearly 40% of global meat production and playing a key role in meeting the dietary needs of rapidly growing urban populations [4,5].

Wet markets are the major retail outlets of poultry and other animal-source foods in many LMICs, as they provide low-cost protein sources and easily accessible options in urban and peri-urban locations [6]. Consumers in wet markets purchase poultry and poultry products that are extremely valued due to their low cost, availability, and freshness. However, even though these markets enable people to afford poultry, they also create a consistent food safety and health risk to the population [7]. Hygiene and handling practices during poultry slaughter and processing in wet markets can contribute to contamination of carcasses with foodborne pathogens such as *Escherichia coli*, *Salmonella*, and *Campylobacter* [8,9]. In many informal market settings, slaughtering activities are conducted within the market environment, where limited cold-chain storage, poor waste disposal, and inadequate sanitation infrastructure increase the risk of microbial contamination. These risks are further amplified by consumer preference for freshly slaughtered poultry, which often results in on-site processing with minimal inspection or protective protocols [10].

The development of antimicrobial resistance (AMR) in foodborne pathogens is considered a major global public health concern [11,12]. In poultry production systems, the widespread use of antibiotics can contribute to the emergence antimicrobial-resistant bacteria, which may spread along the poultry supply chain and through slaughter and handling practices in wet markets [13,14]. For example, multidrug-resistant *Salmonella* has been recovered from carcass rinse water and knives in poultry stalls within wet markets in Dhaka, Bangladesh [14]. In Hong Kong, chicken meat sold in wet markets showed a high prevalence of Extended-Spectrum Beta-Lactamase (ESBL) producing *Escherichia coli*, and from the study, it was observed that there was a lack of compliance with hygiene and food safety regulations among vendors [15]. These findings indicate that antimicrobial-resistant bacteria are present throughout wet market environments and can readily contaminate vendors, consumers, and the food supply chain [14]. Despite growing concern regarding food safety risks in wet markets, limited research has examined the behavioral drivers of food safety practices among poultry vendors operating in urban wet markets in Kenya. Previous studies have largely focused on pathogen contamination and antimicrobial resistance in poultry products, with less attention given to the knowledge, attitudes, and practices of vendors who directly handle and process poultry within these markets. Understanding how vendor characteristics and socioeconomic factors influence food safety behavior is essential for designing effective interventions in informal market systems.

This study aimed to assess the food safety knowledge, attitudes, and practices of poultry vendors in Burma Market, Nairobi, and to examine how vendor demographic and socioeconomic characteristics influence behaviors that may contribute to microbial contamination in this informal wet market setting.

## Materials and methods

### Study design

The study employed a cross-sectional study design targeting poultry vendor in Burma Market and was conducted between 1 July 2025 and 31 October 2025 to assess their level of food safety knowledge, attitudes, and practices.

### Study site

The survey was conducted in Burma Market, a large wet market dealing with poultry, located in Kamukunji Sub-county, Nairobi County, Kenya. Nairobi City was selected for this study because it is Kenya’s largest and fastest‑growing urban center. It has an estimated population exceeding 4 million people [16]. It has a high urban population density of over 6,200 people per square kilometer [17]. Burma Market was also chosen due to its central and easily accessible location and its role as a major poultry trading site where live birds from multiple suppliers are sold in cages and slaughtered on site, creating multiple opportunities for contamination and cross-contamination along the poultry value chain.

### Study population

The study population comprised poultry vendors operating in Burma Market who sold live birds and housed them in cages within the market. Vendors typically owned between one and six cages, with each cage holding approximately 50 birds. The vendors kept the birds alive until sale, either selling them directly to customers or sending them to on-site market slaughterers.

### Inclusion and exclusion criteria

The inclusion criteria were poultry vendors at Burma Market who housed their chickens in cages, had slaughtering activities done primarily within the market setting, were aged 18 years and above, and were willing to provide informed consent. The exclusion criteria were vendors who sold other meats and those that declined to provide informed consent, or operated without caged housing systems.

### Sample size

Convenience sampling was used to recruit study participants. Eligible poultry vendors who were available at the market during the data collection period and consented to participate were enrolled in the study until the desired sample size was achieved. The sample size was calculated using Cochran’s formula for finite populations, assuming a 95% confidence level, a 5% margin of error, and maximum variability (p = 0.5). Based on an estimated vendor population of 200 in Burma Market, the minimum sample size required was 132 vendors. To account for potential non-response, a total of 141 poultry vendors were surveyed.

### Data collection

Data was collected using a semi-structured questionnaire that was administered using the Open Data Kit (ODK) platform. The questionnaire was pre-tested with 20 poultry sellers to assess clarity, flow, and relevance.

### Scoring criteria

Knowledge on hazard entry points, sources of contamination, symptoms of food-borne illnesses, signs of sick birds, and foodborne hazards was assessed using Likert-scale questions. Overall food safety knowledge, attitude, and practices were assessed using 22-item, 5-item, and 8-item questionnaires, respectively. Scores were calculated using a five-point Likert scale and categorized using an 80% threshold based on Bloom’s cut-off point method. The 80% threshold for categorizing knowledge, attitude, and practice scores was adopted based on the established Bloom’s cut-off point method commonly used in KAP studies [18,19]. Detailed scoring ranges and classification criteria are presented in Table 1.

**Table 1:** Scoring of constructs.

Construct	Number of questions	Likert scale	Cut off points	Score range	Categories
Knowledge on hazard entry point	5	5 points	80% (20)	20-25	Knowledgeable
				5-19	Not knowledgeable
Knowledge on sources of contamination	5	5 points	80% (20)	20-25	Knowledgeable
				5-19	Not knowledgeable
Knowledge on signs of sick birds	4	5 points	80% (16)	16-20	Knowledgeable
				4-15	Not knowledgeable
Knowledge on symptoms of food borne illnesses	5	5 points	80% (20)	20-25	Knowledgeable
				5-19	Not knowledgeable
Knowledge on food borne hazards	3	5 points	80% (12)	12-15	Knowledgeable
				3-11	Not knowledgeable
Overall food safety knowledge	22	5 points	80% (88)	88-110	Knowledgeable
				22-87	Not knowledgeable
Attitude	5	5 points	80% (20)	20-25	Positive
				5-19	Negative
Practices	8	5 points	80% (32)	32-40	Good
				8-31	Poor

### Ethical considerations

Ethical clearance for this study was received from Kenyatta National Hospital-University of Nairobi, Ethics and Research Committee (KHN-UoN ERC), reference number P297/03/2025. Permission to conduct the study was sought from the National Commission for Science, Technology& Innovation (NACOSTI) under license number NACOSTI/P/25/4174739. Written informed consent was sought from all participants before administering the study.

### Data analysis

Data collected through the Open Data Kit (ODK) platform were downloaded in Microsoft Excel and imported into Stata version 17 for statistical analysis. Descriptive statistics, including frequencies and percentages, were used to summarize vendors’ socio-demographic characteristics and their food safety knowledge, attitudes, and practices. Associations between categorical variables were assessed using chi-square tests. Multivariate logistic regression analysis was used to examine the influence of socio-demographic factors on food safety knowledge, attitudes, and practices as well as the influence of knowledge and attitude on food safety practices. Adjusted odds ratios (OR) with 95% confidence intervals (CI) were reported, and statistical significance was considered at p < 0.05.

## Results

### Socio-demographic characteristics and operational characteristics of vendors

The majority of vendors were male (58.2%) and aged between 35 and 44 years, with 55.3% of the vendors having attained secondary-level education. Poultry vending was the primary source of income for 97.9% of respondents, and 57.4% reported monthly household incomes ranging from KES 20,000–30,000 (USD 154–231). Most vendors were married (64.5%), and 73.8% operated independently. The majority (81.6%) sourced poultry from farms located outside Nairobi. While a high proportion had access to electricity (80.1%), 53.2% reported access to running water, waste disposal systems (53.9%), and cold storage facilities (56.0%). Most vendors (67.4%) operated from temporary structures, with 17.7% working in open-air setups as shown in Table 2.

**Table 2:** Socio-demographic and operational characteristics of vendors (N = 141).

Variable	Frequency (n)	Percentage (%)
**Age**		
18 to 24 years	20	14.2
25 to 34 years	32	22.7
35 to 44 years	52	36.9
Above 45 years	37	26.2
**Gender**		
Female	59	41.8
Male	82	58.2
**Highest level of education**		
No formal education	1	0.7
Primary	36	25.5
Secondary	78	55.3
Tertiary	26	18.4
**Monthly household income**		
Below Ksh. 20,000	13	9.2
Ksh 20,000- 30,000	81	57.4
Above KSH. 30,000	47	33.3
**Main source of income**		
Crop farming	8	5.7
Salaried work	2	1.4
Casual jobs	1	0.7
Small retail	10	7.1
Livestock keeping	5	3.5
Poultry vending	138	97.9
Meat vendor	1	0.7
**Marital status**		
Single	40	28.4
Divorced/separated	6	4.3
Married	91	64.5
Widowed	4	2.8
**Household size**		
1 to 2 members	39	27.7
3 to 5 members	84	59.6
6 to 8 members	16	11.3
**Number of dependents**		
None	32	22.7
1 to 2	60	42.6
3 to 4	40	28.4
More than 5	9	6.4
**Main language**		
Kiswahili	114	80.9
Local language	27	19.1
**How do you operate**		
Employed by someone	26	18.4
Family business	11	7.8
Independent vendor	104	73.8
**Sources of poultry**		
Farms within Nairobi	63	44.7
Farms outside Nairobi	115	81.6
Middlemen/brokers/traders	66	46.8
Own farm	0	0.0
**Access to resources**		
Electricity	113	80.1
Running water	75	53.2
Cold storage/refrigeration	79	56.0
Waste disposal system	76	53.9
**Business structure**		
Open air setup	25	17.7
Permanent structure	21	14.9
Temporary	95	67.4

### Vendor stall and poultry handling practices

Vendors reported varied levels of experience, with most having sold poultry for 1–3 years (29.1%), followed by those with more than 10 years of experience (27.0%). The majority of vendors (95.7%) operated 1–5 cages, with an average of 59 birds per cage, and most (86.5%) reported restocking poultry daily. The majority (85.5%) stored birds overnight, primarily in temporary cages (49.6%) or covered stalls (47.1%). About two-thirds of vendors (65.2%) mixed birds from different suppliers, although over half (56.7%) reported isolating sick or injured birds. Most vendors (75.2%) fed birds commercial feeds during storage. Poultry sales volumes varied, with (39.0%) selling more than 300 birds per week, and most vendors (96.4%) conducted slaughter within the market slaughterhouse. 66.0% of the vendors reportedly slaughtered and sold sick or dead birds, with 27.0% selling these at reduced prices. Slightly less than half of the poultry vendors (48.2%) reportedly inspected birds prior to sale as shown in Table 3.

**Table 3:** Vendor stall and poultry handling practices (N = 141).

Variable	Frequency (n)	Percentage (%)
**Number of years selling chicken**		
< 1 year	12	8.5
1 to 3 years	41	29.1
3 to 5 years	16	11.3
5 to 10 years	34	24.1
>10 years	38	27.0
**Number of cages used**		
1 to 5	135	95.7
**Average number of birds per cage**		
58.85 ± 44.85		
**How often do you restock**		
Daily	122	86.5
weekly	14	9.9
twice per week	1	0.7
Thrice a week	1	0.7
Depends on sale	3	2.1
**Vaccination records provided by supplier**		
No	131	92.9
Yes	10	7.1
**Who sets prices**		
Market demand	31	22.0
Supplier	8	5.7
Vendor	102	72.3
**Average number of birds sold per week**		
1 to 100 birds	21	14.9
100 to 200 birds	31	22.0
200 to 300 birds	34	24.1
More than 300 birds	55	39.0
**Place of slaughter**		
Market slaughterhouse	135	96.4
On site	1	0.7
Separate area	4	2.9
**Storing birds overnight**		
No	20	14.5
Yes	121	85.5
**Structure used to store birds overnight**		
Covered permanent stall	57	47.1
Open air	2	1.7
Temporary cage structure	60	49.6
Others	2	1.7
**Mixing of birds from different suppliers**		
Yes	92	65.2
No	30	21.3
Sometimes	19	13.5
**Isolation of sick or injured birds**		
Birds are not checked for sickness	18	12.8
No	43	30.5
Yes	80	56.7
**Handling of sick or dead birds**		
Discard	22	15.6
Sell at lower prices	38	27.0
Slaughter and sell	93	66.0
Return to supplier	19	13.5
**Feeding of birds in the stall**		
Commercial feed	106	75.2
Do not feed	15	10.6
Kitchen and market scraps	20	14.2
**Transportation of birds to the stall**		
Broker/ middleman	2	1.4
Hired transporter	14	9.9
Vendor	50	35.5
Supplier farm	75	53.2
**Birds held with feed during storage**		
No	19	13.5
Not sure	1	0.7
Yes	121	85.5
**Inspection of birds before sale**		
No	14	9.9
Yes rarely	59	41.8
Yes regularly	68	48.2

### Birds slaughtering, cleanliness, and hygiene practices of birds

Most vendors (92.2%) hired personnel to conduct slaughter within the market, and tool sharing was common (64.5%), with nearly half (48.9%) cleaning tools only at the end of the day. Defeathering practices varied, with 53.9% of the respondents utilizing both dry and wet methods. In cases of wet defeathering, 75.2% of the poultry vendors reheated scalding water throughout the day and shared defeathering spaces and tools (58.9%). Most vendors reportedly avoided placing birds on the ground (75.9%) and used raised surfaces (92.9%), with only 64.5% maintaining separate surfaces for live birds and dressed carcasses. Waste was mainly collected by market cleaners (65.2%). Water for carcass rinsing was primarily stored in containers such as buckets, drums, or jerrycans (61.0%), typically changed once or twice daily (38.3%) or after each batch (32.6%). Consumer concern was evident, with 58.9% reporting frequent safety inquiries and 61.0% receiving customer complaints or feedback as summarized in Table 4.

**Table 4:** Birds slaughtering, cleanliness, and hygiene practices of birds (N = 141).

Variable	Frequency(n)	Percentage (%)
**Individual slaughtering the birds**		
Hired slaughterer	130	92.2
Vendor	11	7.8
**Sharing of slaughter tools with other vendors**		
No	29	20.6
Sometimes	21	14.9
Yes	91	64.5
**Are slaughtering tools cleaned between uses**		
No	9	6.4
Not sure	17	12.1
Yes, after every bird	46	32.6
Yes, at the end of day	69	48.9
**How clean do you consider your slaughter area**		
Slightly dirty	22	15.6
Neither dirty nor clean	52	36.9
Clean	37	26.2
Very clean	30	21.3
**Defeathering method used**		
Both methods used	76	53.9
Dry defeathering	55	39.0
Wet defeathering	10	7.1
**If dry feathering how are feathers removed**		
By hand	137	97.2
Other	4	2.8
**Handling of scalding water for wet feathering**		
Boiled once per day	8	5.7
Changed regularly between batches	18	12.8
Not changed at all	9	6.4
Reheated throughout the day	106	75.2
**Is defeathering done with other vendors**		
Yes, same space but different tools	52	36.9
Yes, same space and same tools	83	58.9
I use a separate area	6	4.3
**Are defeathering tools cleaned between birds**		
No	12	8.5
Sometimes	23	16.3
Yes, after every bird	50	35.5
Yes, once daily	56	39.7
**Are birds placed on the ground during slaughter or defeathering**		
No	107	75.9
Sometimes	13	9.2
Yes	21	14.9
**What surface do you use for defeathering**		
Raised table bench	131	92.9
Wooden board	10	7.1

### Knowledge of food safety among poultry vendors

29.1% of vendors identified the slaughter operation as a high-risk hazard entry point. The majority of vendors (61.0%) were extremely familiar with signs of illness in birds unfit for sale. Regarding the vendor’s knowledge of the sources of contamination, 44.0% strongly agreed that poor storage in the slaughterhouse contributes to poultry products contamination. The majority of the vendors (57.4%) were very unfamiliar with common food-borne pathogens such as *Escherichia coli* and *Salmonella.* However, most poultry vendors strongly agreed on the common symptoms of food-borne illness as shown in Table 5.

**Table 5:** Knowledge on food safety (N = 141).

KNOWLEDGE ON HAZARD ENTRY POINTS	NO RISK n(%)	LOW RISK n(%)	MODERATE RISK n(%)	HIGH RISK n(%)	VERY HIGH RISK n(%)
At farm	26(18.4)	18(12.8)	23(16.3)	34(24.1)	40(28.4)
During transport	20(14.2)	26(18.4)	35(24.8)	33(23.4)	27(19.1)
During slaughter	14(9.9)	17(12.1)	30(21.3)	39(27.7)	41(29.1)
Storage	14(9.9)	15(10.6)	43(30.5)	40(28.3)	29(20.6)
After sale	34(24.1)	18(12.8)	38(27.0)	24(17.0)	27(19.1)
**KNOWLEDGE ON SIGNS OF SICK BIRDS**	**NOT FAMILIAR** **n(%)**	**SLIGHTLY FAMILIAR** **n(%)**	**FAMILIAR** **n(%)**	**VERY FAMILIAR** **n(%)**	**EXTREMELY FAMILIAR** **n(%)**
Unusual discharge	14(9.9)	5(3.5)	8(5.7)	28(19.9)	86(61.0)
Cannot walk or stand	6(4.3)	2(1.4)	18(12.8)	29(20.6)	86(61.0)
Reduced appetite	9(6.4)	19(13.5)	40(28.4)	23(16.3)	50(35.5)
Swollen body parts	18(12.8)	17(12.1)	29(20.6)	25(17.7)	52(36.9)
**KNOWLEDGE ON SOURCES OF CONTAMINATION**	**STRONGLY DISAGREE** **n(%)**	**DISAGREE** **n(%)**	**NEITHER** **n(%)**	**AGREE** **n(%)**	**STRONGLY AGREE** **n(%)**
Fecal matter	20(14.2)	12(8.5)	19(13.5)	42(29.8)	48(34.0)
Intestinal contents	23(16.3)	9(6.4)	15(10.6)	33(23.4)	61(43.3)
Dirty hands	8(5.7)	11(7.8)	50(35.5)	34(24.1)	38(27.0)
Dirty slaughter house table	8(5.7)	13(9.2)	32(22.7)	40(28.4)	48(34.0)
Poor storage in the slaughter	9(6.4)	13(9.2)	21(14.9)	36(25.5)	62(44.0)
**KNOWLEDGE ON MICROBIAL FOOD HAZARDS**	**VERY FAMILIAR** **n(%)**	**FAMILIAR** **n(%)**	**NEUTRAL** **n(%)**	**UNFAMILIAR** **n(%)**	**VERY UNFAMILIAR** **n(%)**
Foodborne illnesses	14(9.9)	17(12.1)	35(24.8)	20(14.2)	55(39.0)
Pathogens (*Salmonella, E. coli*)	22(15.6)	11(7.8)	3(2.1)	24(17.0)	81(57.4)
Microorganisms/germs	19(13.5)	25(17.7)	27(19.1)	33(23.4)	37(26.2)
**KNOWLEDGE ON SYMPTOMS OF FOOD ILLNESS**	**STRONGLY DISAGREE** **n(%)**	**DISAGREE** **n(%)**	**NEITHER** **n(%)**	**AGREE** **n(%)**	**STRONGLY AGREE** **n(%)**
Vomiting	15(10.6)	1(0.7)	12(8.5)	46(32.6)	67(47.5)
Abdominal pain	14(9.9)	3(2.1)	13(9.2)	48(34.0)	63(44.7)
Diarrhoea	3(2.1)	1(0.7)	10(7.1)	44(31.2)	83(58.9)
Fever	16(11.3)	23(16.3)	42(29.8)	18(12.8)	42(29.8)
Dizziness	15(10.6)	35(24.8)	29(20.6)	14(9.9)	48(34.0)

**Fig 1:**
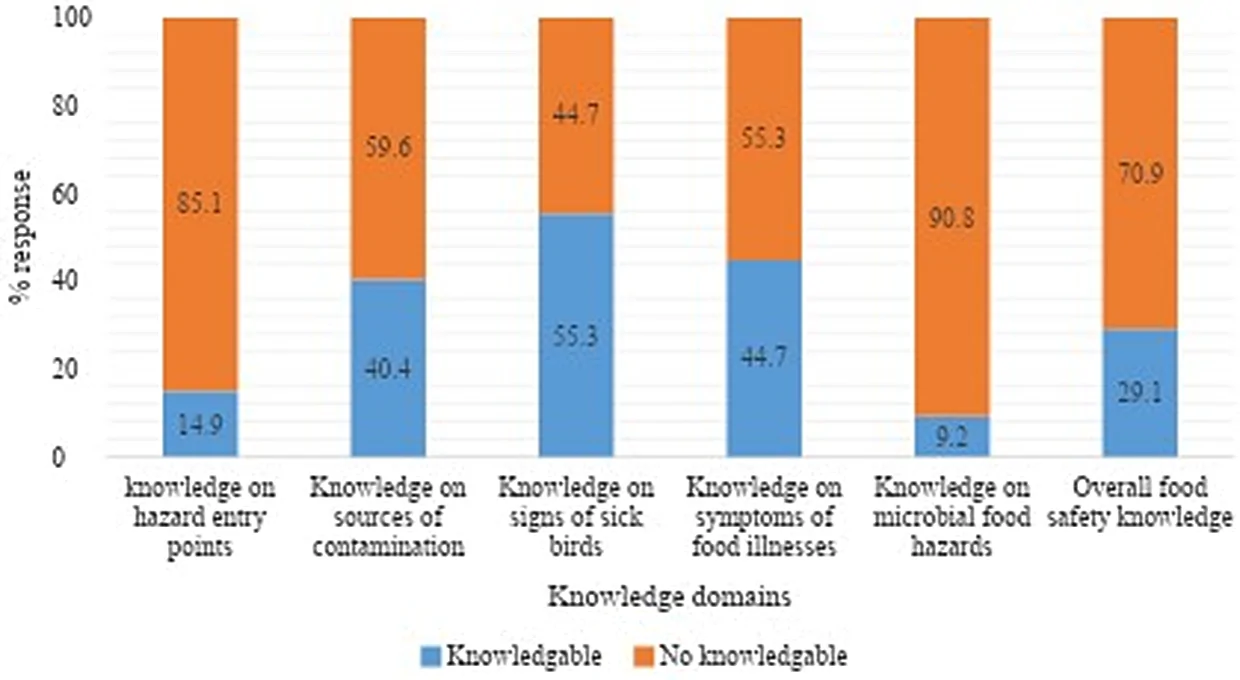
Overall food safety knowledge level among poultry (N = 141).

A majority of vendors were not knowledgeable about hazard entry points (85.1%), sources of contamination (59.6%), symptoms of food-borne illnesses (55.3%), and microbial food hazards (90.8%). However, slightly more than half of the vendors (55.3%) were knowledgeable about signs of sick birds. Overall, most vendors lacked food safety knowledge, while only 29.1% demonstrated adequate food safety knowledge (Fig 1).

### Influence of socio-demographic variables on food safety knowledge

Age was associated with vendors’ food safety knowledge levels. Vendors aged 18–24 years had lower odds of having food safety knowledge compared with those aged 45 years and above (OR = 0.270, 95% CI = 0.029–2.197, p = 0.044). Vendor household income was also associated with food safety knowledge, whereby vendors with a monthly income of KES. 20,000–30,000 had higher odds of having food safety knowledge as compared to those who earned more than KES 30,000 (OR= 3.168, 95% CI = 1.207–8.318, p = 0.019) as shown in Table 6.

**Table 6:** Influence of socio-demographic variables on food safety knowledge.

Variable	Coefficient	Std error	P value	95% CI	
				Lower	Upper
**Age**					
< 45 years	ref				
35-44 years	0.567	0.558	0.309	0.190	1.691
25-34 years	0.414	0.726	0.224	0.100	1.716
18-24 years	0.270	0.521	0.044	0.029	2.197
**Gender**					
Male	ref				
Female	1.131	0.445	0.782	0.473	2.705
**Level of education**					
Tertiary	ref				
Secondary	0.586	0.545	0.326	0.201	1.704
Primary	0.721	0.701	0.641	0.182	2.851
No formal education	1.264	0.987	0.981	0.984	3.875
**Household income**					
Over KES. 30,000	ref				
Ksh. 20,000–30,000	3.168	0.493	0.019	1.207	8.318
Below KES20,000	0.484	0.888	0.414	0.085	2.760
**Marital status**					
Widowed	ref				
Divorced/separated	1.466	1.718	0.824	0.051	42.471
Married	2.231	1.340	0.549	0.161	30.862
Single	2.649	1.383	0.481	0.176	39.847
**Household size**					
Over 8 members	ref				
6-8 members	0.876	0.674	0.651	0.129	1.984
3-5 members	2.341	1.037	0.764	0.932	4.385
1-2 members	1.984	0.987	0.126	0.764	3.095
**Number of dependents**					
Over 5	ref				
3-4	1.837	1.141	0.594	0.196	17.189
1-2	0.845	1.225	0.891	0.077	9.318
None	0.852	1.440	0.912	0.051	14.342

### Food safety attitudes among poultry vendors

A majority of vendors (56.0%) strongly disagreed that preventing contamination is important. Among these, most (54.6%) strongly agreed that it does not matter if consumers become ill, and 42.6% reported being unwilling to change their poultry handling practices, as shown in Table 7.

**Table 7:** Attitude towards food safety.

Statement	Strongly disagree f(%)	Disagree f(%)	Neutral f(%)	Agree f(%)	Strongly agree f(%)
I am not willing to change handling practices	9(6.4)	18(12.8)	36(25.5)	18(12.8)	60(42.6)
It doesn’t matter if consumers get sick	11(7.8)	24(17.0)	7(5.0)	22(15.6)	77(54.6)
Protective gear is unnecessary	10(7.1)	33(23.4)	32(22.7)	20(14.2)	46(32.6)
Refrigeration is expensive and not needed	12(8.5)	35(24.8)	42(29.8)	18(12.8)	34(24.1)
It’s important to prevent contamination	79(56.0)	5(3.5)	10(7.1)	26(18.4)	21(14.9)

### Influence of socio-demographic variables on food safety attitude

Vendor’s household income was associated with food safety attitude, whereby vendors with a monthly income of KES. 20,000–30,000 had lower odds of having positive food safety attitudes compared to those who earned more than KES 30,000 (OR= 0.335, 95% CI = 0.125–0.898, p = 0.030). The number of dependents was associated with food safety attitude among the vendors. Vendors who had no dependents had higher odds of having a positive food safety attitude as compared to those with more than five dependents (OR = 2.289, 95% CI = 0.155–3.877, p = 0.047) as summarized in Table 8.

**Table 8:** Influence of socio-demographic variables on food safety attitude.

Variable	Coefficient	Std error	P value	95% CI	
				Lower	Upper
**Age**					
< 45 years	ref				
35-44 years	1.723	0.563	0.334	0.571	5.194
25-34 years	1.506	0.709	0.564	0.375	6.046
18-24 years	5.121	0.900	0.040	0.877	9.903
**Gender**					
Male	ref				
Female	1.676	0.424	0.223	0.730	3.848
**Level of education**					
Tertiary	ref				
Secondary	1.565	0.528	0.397	0.556	4.407
Primary	1.001	0.703	0.897	0.252	3.966
No formal education	2.345	1.295	0.986	0.956	34.982
**Household income**					
Over KES. 30,000	ref				
KES. 20,000–30,000	0.335	0.503	0.030	0.125	0.898
Below KES. 20,000	0.334	0.800	0.171	0.070	1.605
**Marital status**					
Widowed	ref				
Divorced/separated	1.212	1.523	0.900	0.061	23.996
Married	1.358	1.256	0.807	0.116	15.913
Single	0.297	1.427	0.395	0.018	4.871
**Household size**					
Over 8 members	ref				
6-8 members	0.876	0.543	0.123	0.238	2.094
3-5 members	1.234	0.985	0.347	0.785	3.452
1-2 members	0.651	0.290	0.348	0.208	1.547
**Number of dependents**					
Over 5	ref				
3-4	0.431	1.064	0.429	0.054	3.466
1-2	0.330	1.114	0.319	0.037	2.925
None	2.289	1.375	0.047	0.155	3.877

### Food safety practices among poultry vendors

A majority of the vendors (51.8%) reportedly separated raw poultry from ready-to-eat or cooked foods, and reportedly wore protective clothing (48.9%) when handling poultry. With regards to poultry waste disposal, 32.6% of the respondents reported sometimes disposing of poultry waste safely using sealed containers. However, 80.9% of the poultry vendors reported never having any formal food safety training, as shown in Table 9.

**Table 9:** Food safety practices.

Statement	Always f(%)	Often f(%)	Sometimes f(%)	Rarely f(%)	Never f(%)
I always wash my hands after handling raw poultry or waste.	56(39.7)	23(16.3)	41(29.1)	15(10.6)	6(4.3)
I disinfect tools like knives, cutting boards, and containers used with poultry.	22(15.6)	22(15.6)	43(30.5)	33(23.4)	21(14.9)
I wear protective clothing (gloves, apron, boots) when handling poultry.	69(48.9)	20(14.2)	27(19.1)	15(10.6)	10(7.1)
I clean my poultry handling area (work surface, floors, etc.) at least once a day.	45(31.9)	30(21.3)	26(18.4)	26(18.4)	14(9.9)
I separate raw poultry from cooked or ready-to-eat foods to prevent contamination.	73(51.8)	19(13.5)	20(14.2)	18(12.8)	11(7.8)
I dispose of poultry waste (feathers, offals) properly by using a sealed container.	30(21.3)	12(8.5)	46(32.6)	22(15.6)	31(22.0)
I always use clean, potable water for washing poultry and utensils.	55(39.0)	32(22.7)	31(22.0)	10(7.1)	13(9.2)
I receive regular hygiene or food safety training	7(5.0)	3(2.1)	7(5.0)	10(7.1)	114(80.9)

### Influence of sociodemographic characteristics on food safety practices

Vendor age was associated with food safety practices. Vendors who were 18–24 years old had lower odds of having good food safety practices as compared to those who were 45 years and above (OR= 0.193, 95% CI = 0.136–2.327, p = 0.043). Gender was associated with food safety practices, whereby female vendors had lower odds of having good safety practices as compared to males (OR = 0.411, 95% CI = 0.182–0.926, p = 0.032). Vendor household income was associated with food safety practices. Vendors with a household income of below KES. 20,000 had lower odds of having good food safety practices as compared to those with a household income of over KES. 30,000 (OR= 0.254, 95% CI = 0.059–3.153, p = 0.046). Additionally, marital status was associated with food safety practices, whereby single vendors had very high odds of having good food safety practices as compared to those who were widowed (OR = 11.863, 95% CI = 0.821–17.426, p = 0.040), as shown in Table 10.

**Table 10:** Influence of sociodemographic characteristics on food safety practices.

Variable	Coefficient	Std error	P value	95% CI	
				Lower	Upper
**Age**					
< 45 years	ref				
35-44 years	0.776	0.509	0.619	0.286	2.104
25-34 years	0.716	0.665	0.616	0.195	2.638
18-24 years	0.193	0.783	0.043	0.136	2.327
**Gender**					
Male	ref				
Female	0.411	0.415	0.032	0.182	0.926
**Level of education**					
Tertiary	ref				
Secondary	0.951	0.522	0.923	0.342	2.644
Primary	1.178	0.670	0.807	0.317	4.379
No formal education	0.986	0.453	0.349	0.194	2.093
**Household income**					
Over KES. 30,000	ref				
KES. 20,000–30,000	0.735	0.447	0.490	0.306	1.764
Below KES. 20,000	0.254	0.621	0.046	0.059	3.153
**Marital status**					
Widowed	ref				
Divorced/separated	8.311	1.534	0.167	0.411	17.908
Married	1.995	1.261	0.584	0.168	3.631
Single	11.863	1.363	0.040	0.821	17.426
**Household size**					
Over 8 members	ref				
6-8 members	0.986	0.453	0.673	0.126	2.094
3-5 members	1.873	0.702	0.491	0.305	3.186
1-2 members	0.704	0.350	0.822	0.097	1.620
**Number of dependents**					
Over 5	ref				
3-4	3.857	1.000	0.177	0.543	27.403
1-2	2.810	0.995	0.299	0.400	19.758
None	4.199	1.151	0.213	0.439	40.110

### Association between knowledge, attitudes, and food safety practices

There was an association between knowledge of hazard entry points and food safety practices among the vendors (χ² = 6.234, p = 0.013). A majority (92.8%) of vendors who had good food safety practices were not knowledgeable on hazard entry points, while 7.2%were knowledgeable. There was an association between overall food safety knowledge and food safety practices (χ² = 2.132, p = 0.044). 65.2% of vendors had good food safety practices but had no food safety knowledge. Similarly, a majority of vendors who had poor food safety practices were not knowledgeable about hazard entry points, and overall, food safety knowledge. There was an association between attitude and food safety practices (χ² = 3.512, p = 0.041). A majority (66.7%) of vendors displaying good food safety practices had positive attitudes as summarized in Table 11.

**Table 11:** Association between knowledge domains and food safety practices.

Knowledge domains	Food safety practices		χ^2^	P value
	Good f(%)	Poor f(%)		
**Knowledge on hazard entry point**				
Knowledgeable	5(7.2)	16(22.2)	6.234	0.013
Not knowledgeable	64(92.8)	56(77.8)		
**Knowledge on sources of contamination**				
Knowledgeable	28(40.6)	29(40.3)	0.001	0.971
Not knowledgeable	41(59.4)	43(59.7)		
**Knowledge on signs of sick birds**				
Knowledgeable	40(58.0)	38(52.8)	0.384	0.535
Not knowledgeable	29(42.0)	34(47.2)		
**Knowledge on symptoms of food borne illnesses**				
Knowledgeable	31(44.9)	32(44.4)	0.003	0.954
Not knowledgeable	38(55.1)	40(55.6)		
**Knowledge on food borne hazards**				
Knowledgeable	5(7.2)	8(11.1)	0.629	0.428
Not knowledgeable	64(92.8)	64(88.9)		
**Overall food safety knowledge**				
Knowledgeable	24(34.8)	17(23.6)	2.132	0.044
Not knowledgeable	45(65.2)	55(76.4)		
**Attitude**				
Positive	46(66.7)	58(80.6)	3.512	0.041
Negative	23(33.3)	14(19.4)		

### Influence of knowledge domains and attitude on food safety practices among vendors

Vendors’ food safety attitude was associated with food safety practices. Vendors who had a positive attitude had higher odds of having good safety practices as compared to those who had a negative attitude (OR = 2.196, 95% CI = 0.998–4.834, p = 0.0489), as shown in Table 12.

**Table 12:** Influence of knowledge and attitude on food safety practices among vendors.

Variable	Coefficient	Std error	P value	95% CI	
				Lower	Upper
**Food safety knowledge**					
Knowledgeable	ref				
Not knowledgeable	0.702	0.485	0.466	0.271	1.817
**Attitude**					
Negative	ref				
Positive	2.196	0.402	0.049	0.998	4.834

## Discussion

This study assessed the food safety knowledge, attitudes, and practices of poultry vendors operating in an informal urban wet market in Nairobi. The findings showed that a vast majority of vendors had limited food safety knowledge, negative attitudes toward food safety, and inconsistent hygiene practices during poultry handling and slaughter. Socio-demographic factors, including age, gender, and household income, were associated with variations in food safety behaviors. Vendors with positive attitudes toward food safety were more likely to demonstrate better hygiene practices. These findings suggest that improving food safety in informal wet markets requires interventions that address behavioral and socioeconomic factors influencing vendor practices in addition to training.

In this study, poultry vending in Burma Market was predominantly undertaken by men in their economically productive years, with most vendors having attained secondary-level education. Similar patterns have been reported in wet markets in Bangladesh, where poultry vending is largely dominated by male vendors [20]. Poultry vending also served as the primary source of income for nearly all respondents in this study, highlighting the strong economic dependence of vendors on this activity. Comparable findings have been reported in South Africa, where poultry vending was identified as the main livelihood for most market vendors [21]. Such economic reliance on poultry sales may influence vendor decision-making, particularly in situations where financial pressures discourage the disposal of sick birds. Similar livelihood pressures have been documented in the poultry value chain in Burkina Faso, where vendors sometimes sell sick poultry to avoid financial losses [22].

In this study, the majority of poultry vendors sourced birds from farms located outside Nairobi, indicating a highly interconnected poultry supply chain linking rural farms to urban wet markets. Similar supply chain structures have been documented in other poultry market systems, where birds are transported through networks of traders and intermediaries before reaching retail markets [23]. Such trading networks increase opportunities for the introduction, mixing, and spread of pathogens along the poultry value chain. Studies on Kenyan poultry value chains have also reported that multiple actors and limited biosecurity measures create vulnerabilities for disease transmission within these supply systems [24,25]. In the present study, vaccination records were rarely provided by suppliers, which further limits traceability and may hinder rapid response during disease outbreaks. Similar conditions have been associated with the presence of antimicrobial-resistant bacteria in raw poultry products in Kenya [8].

In this study, several infrastructural limitations were observed within the market environment, including limited access to running water, waste disposal systems, and cold storage facilities, as well as the widespread use of temporary stall structures. Such conditions may create environments that favor microbial persistence and cross-contamination during poultry handling and processing. Similar infrastructural challenges have been reported in wet markets in Hong Kong, where poor hygienic maintenance of meat contact surfaces was associated with increased microbial contamination [15]. These findings highlight the importance of adequate sanitation infrastructure in maintaining food safety within informal market settings [26]. Comparable observations have also been reported in wet markets in Tanzania, where inadequate sanitation infrastructure contributed to unhygienic slaughter and storage conditions [10]. Similar evidence from other Asian wet markets shows that congested stalls and poor sanitation practices can increase the risk of disease transmission in market environments [7].

In this study, many vendors stored birds overnight and frequently mixed poultry obtained from different suppliers within the same cages. Such practices may increase the risk of disease transmission among birds by facilitating close contact and pathogen exchange within crowded holding conditions. Similar concerns have been reported in poultry production systems in Northern Vietnam, where livelihood pressures and market demands were found to conflict with disease risk management practices [27]. The mixing of birds from multiple sources in market environments may therefore contribute to the amplification and spread of infectious agents along the poultry supply chain.

In this study, nearly all vendors relied on hired personnel to carry out poultry slaughter within the market. Similar divisions of labor have been reported in poultry market systems in sub-Saharan Africa, where specialized workers perform slaughter while vendors focus primarily on sales and stall management [28]. While the separation of roles can improve operational efficiency, food safety risks may arise in informal market settings where slaughter tools and processing areas are frequently shared among multiple vendors and sanitation controls are limited. Under such conditions, inadequate cleaning of shared equipment may increase the potential for cross-contamination during poultry processing.

In the present study, poultry vendors reported using both wet and dry defeathering methods, and many vendors reheated scalding water throughout the day rather than replacing it regularly. Such practices may increase the risk of microbial contamination during processing. Previous studies have shown that infrequent replacement of scalding water during wet defeathering can increase bacterial contamination of poultry carcasses because the water may accumulate blood, fecal material, and microorganisms during repeated use [29,30]. Similar hygiene deficiencies have also been reported in poultry retail outlets in Nigeria, where inadequate sanitation of processing surfaces compromised the microbial quality of broiler meat [31]. In addition, microorganisms present on feathers can be transferred to the carcass surface during dry plucking when birds are handled manually, further increasing contamination risks [30]. These findings highlight the importance of proper sanitation and water management during poultry processing in wet market environments.

In the present study, waste management practices within the market environment were often inadequate, with offal, feathers, and other poultry by-products not consistently disposed of using proper sanitation procedures. Poor management of slaughter waste may contribute to unhygienic conditions in market environments and increase the risk of microbial persistence and cross-contamination. Similar sanitation challenges have been reported in slaughterhouse facilities in several developing countries, where improper handling of offal and other by-products compromises hygiene standards [10]. In live-bird markets, inadequate disposal of feathers and offal has also been shown to attract scavengers and facilitate the survival and spread of microorganisms [32]. Such conditions may increase the risk of pathogen transmission through contaminated surfaces and contact between live and processed poultry.

The findings of this study also showed that socioeconomic factors influenced food safety behavior among poultry vendors. In particular, vendor household income was associated with variations in food safety attitudes and practices. Similar relationships between economic conditions and food safety behavior have been reported in Mekelle City, Ethiopia, where higher income levels were linked to improved food safety attitudes and practices among food handlers [33,34]. In contrast, some studies have reported that acceptable food safety practices may occur even when knowledge levels are low. For example, a survey conducted in Pakistan found that food handlers demonstrated relatively good food safety practices despite limited knowledge of food safety principles [35]. Another study in Brazil found no significant association between food safety knowledge and self-reported food safety practices among food handlers indicating that knowledge alone may not be sufficient to predict food handling behavior. Practical experience and routine work practices also play an important role in shaping food safety practices [36]. In the present study, positive food safety attitudes were associated with better food safety practices, a finding that aligns with research in Ethiopia showing that food handlers who perceive food safety as important are more likely to comply with hygiene requirements regardless of their technical knowledge [37,38]. This study highlights the need for integrated food safety interventions in informal wet markets. Given that positive food safety attitudes were associated with good hygiene practices, interventions should extend beyond one-off education sessions to continuous vendor training and behavior change programmes that reinforce safe poultry handling and hygiene practices. Intervention efforts should be supported by investment in market infrastructure such as reliable access to clean water, proper handwashing facilities, and proper waste disposal systems. There is a need to strengthen routine food safety inspections and coordinated action among county governments, public health and veterinary authorities, and market management. Such integrated approaches could improve compliance with safe food-handling practices, reduce the risk of foodborne diseases, and enhance consumer protection in Kenya’s informal poultry markets. Limitations of this study were that the food safety knowledge, attitudes, and practices were assessed using self-reported responses, which may have introduced social desirability bias where some vendors may have underreported unsafe practices or overreported desirable food safety behaviors. This may have been influenced by concerns about potential regulatory implications associated with disclosing poor food safety practices. Time pressures and the busy market environment occasionally limited the depth of responses obtained during interviews. Additionally, this study was conducted in a single urban wet market, and the findings may not be representative of all informal wet markets in Kenya. Future studies involving multiple wet markets across different geographical settings are needed to improve the generalizability of these findings. The study design inherently limits causal inference therefore, the findings in the study are interpreted as associative rather than causal.

## Conclusions

This study demonstrates that food safety practices among poultry vendors in informal wet markets are influenced not only by knowledge but also by vendor attitudes and socioeconomic conditions. Vendors with positive food safety attitudes were more likely to adopt safer hygiene practices, highlighting the importance of behavioral factors in shaping food safety outcomes. Interventions aimed at improving food safety in informal wet markets should therefore go beyond knowledge-based training and address structural barriers such as limited infrastructure, economic pressures, and access to sanitation resources. Strengthening both behavioral and environmental conditions within wet markets may help reduce microbial contamination risks along the poultry supply chain.

## Supporting information

S1 File English version of the food safety knowledge, attitudes and practices questionnaire and observation component. (DOCX)
