# Supplementary material for: Food safety dynamics in urban wet markets: knowledge, attitudes, and practices of poultry vendors in Nairobi, Kenya
Source: PLoS One. 2026 Aug 24;21(8):e0356776. doi: 10.1371/journal.pone.0356776 (PMC13502676; doi:10.1371/journal.pone.0356776)
Supplement: Supplementary file 2 [file pone.0356776.s001.docx]

**KAP QUESTIONNAIRE**

**Pre-Screening Questions**

Are you an ex-layer poultry vendor?

- Yes
- No

Do you sell within Burma Market?

- Yes
- No

Do you currently sell from cages at your stall?

- Yes
- No

How many cages do you operate at your stall?

- 1
- 2
- 3
- 4
- 5
- >5

Do you source chickens from:

- Farmers
- Companies (e.g. commercial layer farms)

Who manages poultry at your stall?

- Man
- Woman
- Youth (18–35)
- Other (specify): ____

Are most of the birds slaughtered and dressed on site?

- Yes
- No

Do you sell poultry outside Burma Market?

- Yes
- No

If yes, where?

- Butcheries
- Open Markets
- Brokers
- Other: ____

**PART I: DEMOGRAPHIC INFORMATION**

What is your age group?

- 18–24
- 25–34
- 35–44
- 45+

What is your gender?

- Male
- Female

What is the highest level of education completed?

- No formal education
- Primary
- Secondary
- Tertiary

What is your household income:

- <KES 20,000
- 20–30K
- >KES 30,000

Which is your main source of income?

- Crop farming
- Salaried work
- Casual jobs
- Small retail
- Livestock keeping
- Poultry vending
- Other meat vendor specify
- Other Specify

**What is your marital Status?**
☐ Single
☐ Married
☐ Divorced/Separated
☐ Widowed

**What is your household size (number of people living in the same house):**
☐ 1–2
☐ 3–5
☐ 6–8
☐ More than 8

**How many dependents do you have?**
☐ None
☐ 1–2
☐ 3–4
☐ 5 or more

**Which is the main language spoken at home?**
☐ Kiswahili
☐ English
☐ Local language (specify): ___________

**How many years of experience do you have in poultry vending?**
☐ Less than 1 year
☐ 1–3 years
☐ 4–6 years
☐ More than 6 years

**How do you operate?**
☐ Independent vendor
☐ Employed by someone
☐ Family business

Other Specify

**What is the average number of chickens sold per week?**
☐ 1–10
☐ 11–30
☐ 31–50
☐ Over 50

**Where do you source your poultry from?** (tick all that apply)
☐ Farms within Nairobi
☐ Farms outside Nairobi
☐ Middlemen/brokers/traders
☐ Own farm

**Do you have access to the following? (tick all that apply):**
☐ Electricity
☐ Running water
☐ Cold storage or refrigeration
☐ Waste disposal system

**What is the type of stall/business structure?**
☐ Permanent structure
☐ Temporary
☐ Open-air setup

**PART II: VENDOR STALL AND POULTRY MANAGEMENT**

How many years do you have selling ex-layer chickens?

- <1
- 1–3
- 3–5
- 5–10
- >10

How many cages do you use? ___

What is the average number of birds per cage? ___

How often do you restock?

- Daily
- Weekly
- Other: ___

Are vaccination/health records provided by suppliers?

- Yes
- No

Who sets pricing?

- You
- Supplier
- Brokers
- Market demand
- Other

What is the average number of birds sold per week?

- 1-100
- 100-200
- 200-300
- 300+

Where is slaughter done?

- On-site
- Market slaughterhouse
- Separate area
- Other: ___

**Live Bird Overnight Holding**

Do you store live birds overnight?
☐ Yes
☐ No

If yes, what type of shelter do you use at night?
☐ Covered permanent stall
☐ Temporary cage/structure
☐ Open air
☐ Other: ___________

**Mixing of Stock and Handling of Sick Birds**

Do birds mix with others from different suppliers?
☐ Yes
☐ No
☐ Sometimes

Is there any isolation for sick or injured birds?
☐ Yes — separate cage
☐ No — mixed with others
☐ Birds are not checked for sickness

What do you do with sick or dead birds? *(tick all that apply)*
☐ Discard
☐ Sell at lower price
☐ Slaughter and sell
☐ Return to supplier
☐ Other: ___________

**Feeding & Transport**

What do you feed the birds while in your stall (if any)?
☐ Commercial feed
☐ Kitchen/market scraps
☐ Do not feed
☐ Other: ___________

Who transports the birds to your stall?
☐ Self
☐ Supplier/farm
☐ Broker/middleman
☐ Hired transporter

Are birds held with water or food during storage?
☐ Yes
☐ No
☐ Not sure

**Observation of Illness**

Do you observe any signs of illness in birds before sale?
☐ Yes — regularly
☐ Yes — rarely
☐ No

**Slaughter Practices**

Where is poultry slaughter performed?

- On-site at stall
- Shared market slaughterhouse
- Off-site area
- Other: …….

Do you slaughter the birds yourself or hire someone?
☐ Self
☐ Hired slaughterer
☐ Rotational with others

Are slaughter tools (e.g., knives) shared with other vendors?
☐ Yes
☐ No
☐ Sometimes

Are the tools used for slaughter cleaned between uses?
☐ Yes — after every bird
☐ Yes — at the end of day
☐ No
☐ Not sure

How clean do you consider your slaughter area on a scale of 1-5?(1=very dirty,2=slightly dirty, 3= Neither, 4= clean,5= very clean)

- 1
- 2
- 3
- 4
- 5

**Defeathering (Dry vs. Wet)**

What method is used for defeathering?
☐ Dry defeathering (manual plucking without hot water)
☐ Wet defeathering (scalding followed by plucking)
☐ Both methods used
☐ Not sure

If wet defeathering is used, how is the hot water managed?
☐ Boiled once per day
☐ Reheated throughout the day
☐ Changed regularly between batches
☐ Not changed at all

If dry defeathering is used, how are feathers removed?
☐ By hand
☐ Using blunt object (e.g., knife spine, stick)
☐ Other: ___________

Is the defeathering area shared with other vendors?
☐ Yes — same space and same tools
☐ Yes — same space, but different tools
☐ No — I use a separate area

Are the tools for defeathering (e.g., knives, plucking tools) cleaned between birds?
☐ Yes — after every bird
☐ Yes — once daily
☐ No
☐ Sometimes

Are birds placed on the ground during slaughter or defeathering?
☐ Yes
☐ No
☐ Sometimes

What surface do you use for defeathering?
☐ Raised table/bench
☐ wooden board
☐ Directly on ground
☐ Other: ___________

What water source is used for rinsing slaughtered birds?
☐ Tap water
☐ Stored water (bucket, drum, jerrycan)
☐ None used
☐ Other: ___________

Is the rinsing water changed or refreshed during the day?
☐ Yes — after every batch
☐ Yes — once or twice per day
☐ No — same water used throughout
☐ Not applicable

Are cleaned carcasses placed on the same surfaces used for live birds?
☐ Yes
☐ No
☐ Sometimes

Are feathers and slaughter waste (e.g., offals, blood) collected or disposed of in a specific way?
☐ Dumped in open bins
☐ Collected by market cleaners
☐ Burned or buried
☐ Thrown in nearby drainage
☐ Other: ___________

**Water Use & Hygiene**

What water source is used for cleaning slaughter tools and hands?
☐ Tap water
☐ Stored water (jerrycan, bucket)
☐ No water used
☐ Other: ___________

Do you use soap/disinfectant when cleaning tools?
☐ Always
☐ Sometimes
☐ Never

Is the same surface used for both live birds and dressed carcass?

- Yes
- No

On a scale of 1-5, how important is hygiene to you when selling poultry?(1=not important, 2=slightly important,3=moderately important,4=very important,5=extremely important)

- 1
- 2
- 3
- 4
- 5

**Waste Management**

How are feathers and waste (e.g., offal, blood) disposed of?
☐ Dumped in open bins
☐ Collected by the market authority
☐ Buried or burned
☐ Other: ___________**Customer Interaction & Perception**

Is poultry displayed or presented in a clean manner at your stall?

- Always
- Sometimes
- Rarely
- Never

| Question | Always-1 | Sometimes -2 | Never-3 |
| --- | --- | --- | --- |
| Do customers ever ask about the hygiene or safety of poultry? |  |  |  |
| Do you discuss freshness or cleanliness with customers? |  |  |  |
| Do you receive feedback or complaints about the poultry you sell? |  |  |  |

**PART III: KNOWLEDGE OF HAZARD ENTRY POINTS**

Please rate your understanding of the likelihood of contamination occurring at the following stages(1= No risk,2=low risk, 3=moderate risk,4=high risk,5=very high risk)

| Stage | 1=No risk | 2=low risk | 3=moderate risk | 4=high risk | 5=very high risk |
| --- | --- | --- | --- | --- | --- |
| At farm |  |  |  |  |  |
| During transport |  |  |  |  |  |
| At stall |  |  |  |  |  |
| During slaughter |  |  |  |  |  |
| Storage |  |  |  |  |  |
| After sale |  |  |  |  |  |

Do you monitor poultry health before slaughter/sale?

- Yes
- No

What measures do you take if a chicken seems sick?

- Isolate
- Sell immediately
- Treat
- Return to supplier
- Other: ___

How often do you inspect your cages for hygiene/safety?

- Daily
- Weekly
- Occasionally
- Never

What do you do if your birds show signs of illness?

- Report to the authority
- Self-medicate
- Sell quickly
- Nothing
- Other: ___

**Are birds from different sources kept in the same cage?**
☐ Yes — always
☐ Sometimes
☐ No — kept separate

**Do you observe birds for unusual behaviour (e.g., not eating, drooping, discharge)?**
☐ Yes — regularly
☐ Only if obvious signs
☐ No

**Have you ever received training on identifying sick birds or poultry diseases?**
☐ Yes
☐ No

**Do you know any signs that indicate a bird might be unfit for sale?** (tick all that apply)
☐ Unusual discharge
☐ Cannot stand/walk
☐ Reduced appetite
☐ Swollen body parts
☐ I don’t know

**Do you separate carcasses of birds that died before slaughter from those slaughtered normally?**
☐ Yes
☐ No
☐ I don’t deal with dead birds

**Do you wash or sanitize your cages or holding areas?**
☐ Yes — daily
☐ Weekly
☐ Occasionally
☐ Never

**What do you use to clean cages or stalls?**
☐ Water only
☐ Water + soap
☐ Water + disinfectant
☐ I don’t clean

**Do you use any substances (e.g., herbs, over-the-counter meds) to boost bird health before sale?**
☐ Yes — always
☐ Sometimes
☐ No

**If you treat sick birds, what kind of treatment do you use?** (tick all that apply)
☐ Traditional remedies
☐ Human medicine
☐ Animal-specific drugs
☐ I don’t treat

**Do you keep records of when and how birds are treated or medicated?**
☐ Yes — written
☐ Yes — verbal/memory
☐ No

**Are sick or dead birds ever sold to customers?**
☐ Yes — if still usable
☐ Never
☐ Sometimes (if pressured)
☐ Prefer not to say

**PART IV: FOOD SAFETY KNOWLEDGE**

Please rate how strongly you agree that the following can contaminate poultry meat(1= strongly disagree,5= strongly agree)

| **Contamination source** | **Strongly disagree-1** | **Disagree-2** | **Neither-3** | **Agree-4** | **Strongly agree-5** |
| --- | --- | --- | --- | --- | --- |
| Fecal matter |  |  |  |  |  |
| Intestinal contents |  |  |  |  |  |
| Dirty hands |  |  |  |  |  |
| Dirty slaughter house table |  |  |  |  |  |
| Poor storage slaughter |  |  |  |  |  |

Rate your familiarity with the following signs of illness that may indicate poultry is unfit for sale(1= Not Familiar, 5= Very Familiar)

| **Signs of illness** | **Not familiar-1** | **Slightly Familiar-2** | **Familier-3** | **Very familiar-4** | **Extremely familiar-5** |
| --- | --- | --- | --- | --- | --- |
| Unusual discharge |  |  |  |  |  |
| Cannot walk or stand |  |  |  |  |  |
| Reduced appetite |  |  |  |  |  |
| Swollen body parts |  |  |  |  |  |

Please indicate your agreement with whether the following are symptoms of foodborne illness( 1=strongly disagree,5=strongly agree)

| **Symptom** | **Strongly disagree-1** | **Disagree-2** | **Neither-3** | **Agree-4** | **Strongly agree-5** |
| --- | --- | --- | --- | --- | --- |
| Vomiting |  |  |  |  |  |
| Abdominal pain |  |  |  |  |  |
| Diarrhoea |  |  |  |  |  |
| Fever |  |  |  |  |  |
| Dizziness |  |  |  |  |  |

Is carcass contamination dangerous to consumers?

- Yes
- No

Causes of foodborne illness (tick all where applicable):

- Germs
- Chemicals
- Dirt
- Physical debris
- Other: ___

Which temperate range is poultry meat likely to become unsafe due to bacterial growth?

- 0–4°C
- 5–60°C
- >60°C

Why is boiling scald water important?

- Easier feather removal
- Kills microorganisms
- Cleans contamination
- Improves tenderness
- Other: ___

Familiarity with food safety concepts (score scale 1–5):

| SYMPTOM | Very Familiar- 5 | Familiar - 4 | Neutral-3 | Unfamilier-2 | Very Unfamilier-1 |
| --- | --- | --- | --- | --- | --- |
| Foodborne illnesses |  |  |  |  |  |
| Pathogens(*Salmonella, E. coli*) |  |  |  |  |  |
| Microorganisms/germs |  |  |  |  |  |

**PART V: FOOD SAFETY ATTITUDES**

(Score 1–5: Strongly Agree – Strongly Disagree)

| **Statement** | Strongly disagree -1 | Disagree-2 | Neutral- 3 | Agree-4 | Strongly agree-5 |
| --- | --- | --- | --- | --- | --- |
| I am not willing to change handling practices |  |  |  |  |  |
| It doesn't matter if consumers get sick |  |  |  |  |  |
| Protective gear is unnecessary |  |  |  |  |  |
| Refrigeration is expensive and not needed |  |  |  |  |  |
| It’s important to prevent contamination |  |  |  |  |  |

| Question | Always-1 | Sometimes -2 | Never-3 |
| --- | --- | --- | --- |
| Do you wear gloves when handling poultry? |  |  |  |
| Do you wear an apron or coat while working with poultry? |  |  |  |
| Do you wear a mask while handling poultry or during cleaning? |  |  |  |
| Do you use gumboots or closed shoes while working with poultry? |  |  |  |
| After sale |  |  |  |

How often do you clean PPE?

- After each use
- Daily
- Weekly
- Never

Is there a designated clean slaughter area?

- Yes
- No

Do you wash hands before/after handling poultry?

- Always
- Sometimes
- Never

Do you use clean water for poultry and utensils?

- Always
- Sometimes
- Never

How often do you change scalding water?

- Every batch
- Once daily
- Rarely

Have you attended food safety training?

- Yes
- No

Who provided the training?....................

Would you attend future training?

- Yes
- No
- Maybe

**Is PPE (e.g., gloves, apron, boots) available at all times at your work area?**
☐ Yes
☐ No

**If PPE is unavailable, what do you use instead?**
☐ Normal clothing
☐ Makeshift protective gear
☐ Nothing
☐ Other: ___

**Where do you store your PPE after use?**
☐ In a clean storage area
☐ Anywhere available
☐ At home
☐ I don’t store PPE

**Do you believe the market authority or government checks your hygiene practices?**
☐ Yes — regularly
☐ Rarely
☐ Never
☐ I don’t know

**What challenges prevent you from following better hygiene practices?** (tick all that apply)
☐ Water shortage
☐ No proper slaughter space
☐ Time pressure
☐ Cost of PPE/cleaning
☐ Lack of training
☐ Market conditions (dust, congestion)

| Statement | 1=Always | 2=Often | 3=Sometimes | 4=Rarely | 5=Never |
| --- | --- | --- | --- | --- | --- |
| I always wash my hands after handling raw poultry or waste. |  |  |  |  |  |
| I disinfect tools like knives, cutting boards, and containers used with poultry. |  |  |  |  |  |
| I wear protective clothing (gloves, apron, boots) when handling poultry. |  |  |  |  |  |
| I clean my poultry handling area (work surface, floors, etc.) at least once a day. |  |  |  |  |  |
| I separate raw poultry from cooked or ready-to-eat foods to prevent contamination. |  |  |  |  |  |
| I dispose of poultry waste (feathers, offals) properly by using a sealed container. |  |  |  |  |  |
| I always use clean, potable water for washing poultry and utensils. |  |  |  |  |  |
| I receive regular hygiene or food safety training |  |  |  |  |  |

**ENUMERATOR OBSERVATIONS**

**Stall & Infrastructure**

☐ Permanent / ☐ Temporary / ☐ Open-air stall

☐ Covered from dust and rain

☐ Access to clean water container

☐ Soap/disinfectant available

☐ Market drainage system nearby

**Live Bird Management**

☐ Cages overcrowded

☐ Cages elevated (off ground)

☐ Sick/dead birds visible

☐ Separate cages for different sources

☐ Sick birds isolated or not

**Slaughter & Defeathering**

☐ Slaughter done on site

☐ Shared slaughter tools with others

☐ Tools cleaned after each bird / daily / not at all

☐ Dry plucking observed

☐ Wet defeathering observed

☐ Scalding water reused or replaced

☐ Slaughter on ground or clean surface

☐ Hands washed between birds

**Personal Hygiene & PPE**

PPE Worn:

☐ Apron

☐ Gloves

☐ Hairnet/cap

☐ Boots

Hands washed during activity:

☐ Yes

☐ No

**Sanitation & Waste Handling**

☐ Feathers and blood disposed in proper bin

☐ Waste scattered on ground/drainage

☐ Clean water used for rinsing carcasses

☐ Carcasses handled on clean trays/boards

☐ Carcasses placed on ground: ☐ Yes ☐ No

**Training & Records (Visual Cues)**

☐ Any poster or food safety sign at stall

☐ Health/vaccination records shown by vendor

☐ Presence of training certificate or market rules displayed

**Customer Interaction**

☐ Vendor advises customers about freshness/hygiene

☐ Clean presentation of carcasses (no feathers, blood)

☐ Customers ask about hygiene:

☐ Yes

☐ No

**THANK YOU FOR YOUR PARTICIPATION!**
