# Supplementary material for: Food safety dynamics in urban wet markets: knowledge, attitudes, and practices of poultry vendors in Nairobi, Kenya
Source: PLoS One. 2026 Aug 24;21(8):e0356776. doi: 10.1371/journal.pone.0356776 (PMC13502676; doi:10.1371/journal.pone.0356776)
Supplement: Supplementary file 3 [file pone.0356776.s002.docx]

Major Comments

1. Study design and methodology:

The cross-sectional design is appropriate for assessing KAP; however, it limits causal inference and this should be more clearly emphasized. The sampling approach is not clearly described, raising concerns about potential selection bias.

2. Measurement and scoring system:

The use of an 80% cutoff to define 'good' vs 'poor' knowledge, attitude, and practice appears arbitrary and requires justification. Variation in the number of questions across constructs may introduce measurement bias.

3. Statistical analysis:

Some reported odds ratios have confidence intervals close to or crossing 1 but are interpreted as significant. Clarification is needed on whether multivariable regression was performed and which confounders were adjusted for.

4. Interpretation of results:

Some conclusions appear overstated, particularly regarding the role of attitudes. The finding that good practices can occur despite low knowledge should be further explored.

5. Validity of self-reported data:

The observational component is mentioned but not clearly integrated into analysis. Potential social desirability bias should be more critically discussed.

Minor Comments

Language is generally clear but could be improved for conciseness. Tables should be checked for consistency and clarity. Minor formatting issues are present.

Ethics and Transparency

Ethical approval and informed consent are clearly stated. Data availability complies with journal requirements. No major ethical concerns identified.

Recommendation

Major Revision

The study addresses an important issue but requires methodological clarification and improved statistical interpretation before publication.
