# Supplementary material for: Food safety dynamics in urban wet markets: knowledge, attitudes, and practices of poultry vendors in Nairobi, Kenya
Source: PLoS One. 2026 Aug 24;21(8):e0356776. doi: 10.1371/journal.pone.0356776 (PMC13502676; doi:10.1371/journal.pone.0356776)
Supplement: Supplementary file 4 [file pone.0356776.s004.docx]

Lolyne Kaimenyi
Department of Food Science, Nutrition and Technology
University of Nairobi
Nairobi, Kenya

2 July 2026

The Chief Editor
PLOS ONE

Dear Editor,

**Subject: Manuscript Resubmission**

Thank you for the valuable comments and feedback on our manuscript. We have carefully addressed all the comments raised and revised the manuscript accordingly. Please find attached the reviewers’ comments, our responses to the comments for our manuscript entitled, “Food safety dynamics in urban wet markets: knowledge, attitudes, and practices of poultry vendors in Burma Market, Nairobi, Kenya- PONE-D-26-11826”, for consideration for publication in PLOS ONE.

|  | **COMMENTS** | **RESPONSE** |
| --- | --- | --- |
| 1. | The cross-sectional design is appropriate for assessing KAP; however, it limits causal inference and this should be more clearly emphasized. | Thank you for your insightful comment regarding the limitations of casual inference in our cross-sectional design. A statement has been added to the discussion to indicate the limitation of the study design to read indicating that the findings in the study were interpreted as associative rather than causal.  Page 24, Line 378-380 |
| 2. | The sampling approach is not clearly described, raising concerns about potential selection bias. The sampling strategy/approach is not clearly described.  [Similar comment] Clarification is needed on whether the sample was random or convenience-based, as this has implications for selection bias. | A sampling technique has been added on convenience sampling was used to recruit study participants. Eligible poultry vendors who were available at the market during the data collection period and consented to participate were enrolled in the study until the desired sample size was achieved. Page 5, Line 110-112 |
| 3. | The use of an 80% cutoff to define 'good' vs 'poor' knowledge, attitude, and practice appears arbitrary and requires justification. Variation in the number of questions across constructs may introduce measurement bias.  [Similar comment] The use of an 80% cutoff to classify knowledge, attitudes, and practices is not justified. The authors should provide a rationale or supporting references. | Reference of previous use of the 80% threshold has been provided in the reference used for the methodology and has been cited “The 80% threshold for categorizing knowledge, attitude, and practice scores was adopted based on the established Bloom's cut-off point method commonly used in KAP studies [18,19].”  References added in Page 6, Line 126-128 and, Page 28 & 29, Line 479-487 |
| 4. | Some reported odds ratios have confidence intervals close to or crossing 1 but are interpreted as significant. Clarification is needed on whether multivariable regression was performed and which confounders were adjusted for.  [Similar comment] Some reported odds ratios have confidence intervals that approach or cross 1, yet are interpreted as significant. The authors should carefully review statistical interpretations and clarify whether multivariable analyses were performed. | Data was subjected to Multivariate binary regression analysis and this has been updated in the manuscript to read “Multivariate logistic regression analysis was used to examine the influence of socio-demographic factors on food safety knowledge, attitudes, and practices as well as the influence of knowledge and attitude on food safety practices. Adjusted odds ratios (OR) with 95% confidence intervals (CI) were reported, and statistical significance was considered at p < 0.05.” Page 7, Line 143-147  The statistical interpretation of results reported as significant has been reviewed and revised throughout the document. |
| 5. | Some conclusions appear overstated, particularly regarding the role of attitudes.  [Similar comment] Some conclusions are slightly overstated, particularly regarding the relative importance of attitudes versus knowledge. | The statement on attitude was rewritten to read “There was a significant association between attitude and food safety practices (χ² = 3.512, p = 0.041). A majority (66.7%) of vendors displaying good food safety practices had positive attitudes as summarized in Table 11” as outlined in Page 18 &19, Line 263-265 |
| 6. | The discussion should better reflect the limitations of a cross-sectional design. | The statement has been added to address the limitation “The study design inherently limits causal inference therefore, the findings in the study are interpreted as associative rather than causal.’’ Page 24, Line 378-380 |
| 7. | The finding that good practices can occur despite low knowledge should be further explored | Supporting study was added in the discussion “Another study in Brazil found no significant association between food safety knowledge and self-reported food safety practices among food handlers indicating that knowledge alone may not be sufficient to predict food handling behavior. Practical experience and routine work practices also play an important role in shaping food safety practices” in Page 23, Line 365-369 and references added in Page 31, Line 549-552 |
| 8. | The observational component is mentioned but not clearly integrated into analysis.  [Similar comment] Although an observational component is mentioned, it is not clearly integrated into the analysis. This should be clarified or strengthened. | The observational study section was removed. |
| 9. | The discussion of limitations should be expanded, especially regarding potential social desirability bias and generalizability.  [Similar comment] Potential social desirability bias should be more critically discussed. | It has been addressed in the limitations to read “Limitations of this study were that the food safety knowledge, attitudes, and practices were assessed using self-reported responses, which may have introduced social desirability bias where some vendors may have underreported unsafe practices or overreported desirable food safety behaviors. This may have been influenced by concerns about potential regulatory implications associated with disclosing poor food safety practices.” Page 24, Line 372- 377 |
| 10. | Please review your reference list to ensure that it is complete and correct. If you have cited papers that have been retracted, please include the rationale for doing so in the manuscript text, or remove these references and replace them with relevant current references. Any changes to the reference list should be mentioned in the rebuttal letter that accompanies your revised manuscript. If you need to cite a retracted article, indicate the article’s retracted status in the References list and also include a citation and full reference for the retraction notice. | The reference list was reviewed and no retracted references were identified. Minor corrections were made on formatting. |
| 11. | Improve clarity and reduce repetition in some sections. | The manuscript was revised to improve clarity and flow. The scoring criteria section was condensed to avoid repetition from data presented in Table 1.  The introduction and abstract sections were refined.  Study population section was revised to clarify participant eligibility and the discussion section was revised. Edits were also made throughout the manuscript to improve consistency. |
| 12. | Ensure consistency in tables and formatting | All tables were reviewed for consistency in formatting, labeling, and presentation. |
| 13. | Please ensure that your manuscript meets PLOS ONE's style requirements, including those for file naming | Corrections were been made on file naming and author affiliation details to match PLOS 1 requirements. |
|  | **OTHER COMMENTS WITHIN THE MANUSCRIPT** | |
| 14. | ‘Estimated vendor population of 200 in Burma Market’  Is this the estimated population of vendors that met the inclusion criteria? or all vendors in the market? | The estimated population refers to all poultry vendors operating within Burma Market at the time of the study. This has been addressed to read “ Based on an estimated vendor population of 200 in Burma Market, the minimum sample size required was 132 vendors. ” Page 5, Line 114-115 |
| 15. | Study population  ‘ready poultry products’  What does this mean? Ready to cook  ? | This has been corrected to read “The study population comprised poultry vendors operating in Burma Market who sold live birds and housed them in cages within the market. Vendors typically owned between one and six cages, with each cage holding approximately 50 birds” Page 5, Line 98-100 |
| 16. | Study population  So, were the live birds slaughtered by someone else within the market itself? In other words, did both the sale of live birds and the slaughtering process take place within the market? | The sale and slaughter of live birds took place within the market.  This has been corrected to read “The vendors kept the birds alive until sale, either selling them directly to customers or sending them to on-site market slaughterers.  .” Page 5, Line 100-102 |

Yours sincerely,

Lolyne Kaimenyi
