# Supplementary material for: Food safety dynamics in urban wet markets: knowledge, attitudes, and practices of poultry vendors in Nairobi, Kenya
Source: PLoS One. 2026 Aug 24;21(8):e0356776. doi: 10.1371/journal.pone.0356776 (PMC13502676; doi:10.1371/journal.pone.0356776)
Supplement: Supplementary file 5 [file pone.0356776.s005.docx]

Lolyne Kaimenyi
Department of Food Science, Nutrition and Technology
University of Nairobi
Nairobi, Kenya

24 July 2026

The Chief Editor
PLOS ONE

Dear Editor,

**Subject: Manuscript Resubmission**

Thank you for the valuable comments and feedback on our revised manuscript. We have carefully addressed all the comments raised and revised the manuscript accordingly. Please find attached the reviewers’ comments, our responses to the comments for our manuscript entitled, “Food safety dynamics in urban wet markets: knowledge, attitudes, and practices of poultry vendors in Burma Market, Nairobi, Kenya- PONE-D-26-11826R1”, for consideration for publication in PLOS ONE.

|  | **COMMENTS** | **RESPONSE** |
| --- | --- | --- |
| 1. | Check and correct the logistic regression results.  Statistics analyses are mostly suitable, but authors are urged to scrutinize logistic regression output. Certain odds ratios, confidence intervals, and associated p values in Table 3 are contradictory (e.g. CI including 1, but a significant p-value reported). These numbers must be checked and corrected if applicable. | The logistic regression results were carefully reviewed against the original statistical output. The odds ratios, 95% confidence intervals, and p-values were verified, and any transcription inconsistencies identified during the review were corrected. The revised values are reflected in the following sections:  Abstract – Page 1, Lines 19–20 and 26  Table 6 – Page 15, Lines 210 and 214  Table 8 – Page 16, Line 231  Table 10 – Page 18, Lines 244, 249 and 253  Confounders were not included in the regression model. Chi square was done between knowledge domains and practices while regression model was for overall knowledge, attitude and practices. |
| 2. | A bit stronger comment in Discussion on the policy and public health implications is warranted. Could add one paragraph highlighting how policy and public health interventions should be adapted to the findings in the context of the informal wet market context. | This has been addressed and the following paragraph has been added ‘‘This study highlights the need for integrated food safety interventions in informal wet markets. Given that positive food safety attitudes were associated with good hygiene practices, interventions should extend beyond one-off education sessions to continuous vendor training and behaviour change programmes that reinforce safe poultry handling and hygiene practices. Intervention efforts should be supported by investment in market infrastructure such as reliable access to clean water, proper handwashing facilities, and proper waste disposal systems. There is a need to strengthen routine food safety inspections and coordinated action among county governments, public health and veterinary authorities, and market management. Such integrated approaches could improve compliance with safe food-handling practices, reduce the risk of foodborne diseases, and enhance consumer protection in Kenya's informal poultry markets.’’  Page 24, Line 372-382 |
| 3. | The discussion on limitations seems adequate, authors may need to briefly reflect that generalizability to other locations is somewhat limited as study was conducted in only one urban wet market. | This has been addressed and the following paragraph has been added ‘‘Additionally, this study was conducted in a single urban wet market, and the findings may not be representative of all informal wet markets in Kenya. Future studies involving multiple wet markets across different geographical settings are needed to improve the generalizability of these findings.’’  Page 24, Line 388- 391 |

Yours sincerely,

Lolyne Kaimenyi
